# Effectiveness of screening for tuberculosis in HIV: a pragmatic clinical trial

**DOI:** 10.11606/s1518-8787.2021055002936

**Published:** 2021-07-19

**Authors:** Marcela Lopes Santos, Joanna d’Arc Lyra Batista, Cynthia Braga, Adriana Paula da Silva, Magda Maruza, Wayner Vieira Souza, Maria Rosimery de Carvalho, Noemia Teixeira de Siqueira-Filha, Maria de Fátima Pessoa Militão de Albuquerque

**Affiliations:** I Instituto Aggeu Magalhães Departamento de Saúde Pública RecifePE Brasil Instituto Aggeu Magalhães. Departamento de Saúde Pública. Recife, PE, Brasil.; II Universidade Federal da Fronteira Sul Faculdade de Medicina ChapecóSC Brasil Universidade Federal da Fronteira Sul. Faculdade de Medicina. Chapecó, SC, Brasil.; III Hospital Correia Picanço RecifePE Brasil Hospital Correia Picanço. Recife, PE, Brasil.; IV University of York Department of Health Sciences York UK University of York. Department of Health Sciences. York, UK.

**Keywords:** HIV Infections, Tuberculosis, diagnosis, Mass Screening, Clinical Trial

## Abstract

**OBJECTIVE:**

To verify the effectiveness of screening for tuberculosis (TB) on all-cause mortality and tuberculosis cases in newly diagnosed HIV-infected patients through a clinical algorithm based on recommendations of the World Health Organization.

**METHODS:**

From March 2014 to April 2016, a pragmatic randomized clinical trial was conducted with newly diagnosed and TB-free HIV-infected adults undergoing antiretroviral therapy for up to one month at a major tertiary hospital for HIV in the state of Pernambuco, Brazil. Participants were randomized into intervention and control groups using an automatically-generated random list, and followed-up for at least 6 months. The intervention group was screened for TB at hospital admission and at every follow-up visit through a series of questions addressing TB-related symptoms (cough, fever, night sweating, and weight loss). Patients presenting with any of these symptoms were referred to a pulmonologist and underwent sputum smear microscopy, sputum culture, and rapid molecular testing (GeneXpert). When at least one test result came back positive, TB treatment was initiated. In turn, if patients tested negative but presented with severe clinal symptoms, TB preventive treatment was initiated. Screening for TB was not performed systematically in the control group. The primary outcome assessed in this study was death from all causes, and secondary outcomes included sensitivity and specificity of this screening test, as well as its detection time.

**RESULTS:**

This study evaluated 581 patients, 377 in the intervention group (64.9%) and 204 in the control group (35.1%). In total, 36 patients died during the follow-up period. Of these, 26 (6.9%) were from the intervention group, reaching a cumulative mortality coefficient of 69 per 1,000 inhabitants, and 10 (4.9%) from the control group (p = 0.341), with a cumulative mortality coefficient of 49 per 1,000 inhabitants (p = 0.341).

## INTRODUCTION

The synergy between the human immunodeficiency virus (HIV) and Mycobacterium tuberculosis has been a major threat for public health due to its magnitude and the high mortality rates from tuberculosis (TB) among people living with HIV (PLHIV). Nowadays, about 10% of TB cases worldwide are estimated to occur among PLHIV, while 22% of deaths due to TB are attributed to TB-HIV coinfection^1^. Studies have shown that an early diagnosis and treatment for tuberculosis may help reducing such rate among PLHIV^2^^,^^3^. The negative outcomes related to tuberculosis are enhanced before co-infection with TB-HIV, often resulting from late diagnosis due to the lower sensitivity of diagnostic tests (low bacilli load in biological samples) and poor treatment response^4^^–^^7^.

According to the World Health Organization (WHO), screening for TB in PLHIV should be performed through a clinical algorithm addressing symptoms often related to the condition, such as cough of any duration, fever, night sweats, and weight loss. Patients presenting at least one of these symptoms must be further investigated by undergoing specific tests^8^. However, recent studies have presented inconclusive results depending on the region where they are performed^9^^–^^17^. In this sense, our study aims is to verify the effectiveness of a screening test for TB performed through a clinical algorithm among PLHIV in the routine of a specialized health service in HIV, verifying its impact on all-cause mortality.

## METHODS

### Trial Design

This is a pragmatic randomized clinical trial conducted in a public health service in Recife/Pernambuco. To verify the real impact of the intervention, this design is recommended for studies evaluating the routine of care teams in health services. The intervention group was attended by five doctors and nurses trained for TB screening. Individuals were allocated to the intervention group in a 2:1 ratio in relation to the control group.

This study was conducted according with the CONSORT guidelines for pragmatic trials.

### Changes to Methods after Trial Commencement

As the hospital where our trial was conducted was the major service for HIV/AIDS in the state of Pernambuco, our study considered a larger sample at first. However, the decentralization policy enabled patients to receive care in health services near their residences, so that the number of newly diagnosed HIV-positive patients drastically reduced in the service in question. To achieve the research objectives, we had to redesign the sample.

### Participants

Our study population comprised newly diagnosed HIV-positive adult patients with either no previous record of or one-month antiretroviral therapy (ART), and with no history of diagnosis or treatment of TB during the past three months. These patients were treated and recruited between March 2014 and April 2016 in the outpatient clinic of the Correia Picanço Hospital (CPH) – a referral center for people living with HIV responsible for treating around 50% of AIDS and HIV-infected individuals in the Pernambuco state, Northeastern Brazil. Participants were treated by spontaneous demand or referred from other health services or from CPH emergency department and ward. Incarcerated patients were excluded due to the difficulties in follow-up.

### Interventions

Trained nurses administered a standardized questionnaire to both groups (control and intervention) at hospital admission, collecting socioeconomic and lifestyle data, as well as information on previous TB episodes. As recommended by the WHO, a second questionnaire was applied to collect data on symptoms related to tuberculosis, such as cough, fever, night sweats, and weight loss. Participants presenting with at least one of these symptoms were referred to a pneumologist and underwent TB investigation through clinical assessment and chest X-ray (before the impossibility of providing at least 2 mL of sputum) or sputum smear microscopy, sputum culture, and GeneXpert MTB/RIF (when patients were able to provide a one-spot sputum of at least 2mL).

Patients with at least one positive result for sputum smear test or GeneXpert MTB/RIF were classified as bacteriologically confirmed TB cases and initiated treatment for TB. In turn, patients with chest X-ray results indicative of TB and/or presenting criteria for clinical severity (anemia, CD4+ T-cell lymphocyte count < 200 cells/mm3, and BMI < 18,5), were classified as presumptive TB and initiated empirical therapy. All patients undergoing treatment for TB were followed up by both a pneumologist, responsible for assessing the progress of tuberculosis treatment, and by an infectologist, responsible for assessing the progress of HIV/AIDS treatment.

Patients in the intervention group who presented with no tuberculosis-related symptoms at screening and those whose tests results came back negative for both confirmed or presumptive TB were followed up by an infectologist and underwent the same clinical screening for TB at every follow-up visit. Considering that the health status of these patients could eventually deteriorate, leading them to search for care in other services where they could be diagnosed with TB or even die, we consulted the medical records made available by other health services in search for these cases.

The control group comprised patients following the routine of the Health Service, so that the disease investigation was left to the discretion of physicians.

### Outcomes

The primary outcome evaluated in our study was death from all causes, and secondary outcomes included tuberculosis cases. Outcomes were obtained by probabilistic linkage between data collected in our research and mortality records related to Pernambuco, obtained from the Mortality Information System (SIM). TB cases were identified by establishing an association between our data and that from the Information System for Notifiable Disease (Sinan).

### Sample Size

Sample size was calculated based on the proportion of deaths between the exposed (intervention) and the non-exposed group (control). Determined from the 2014 approximate mortality rate due to HIV in Pernambuco (6.2%)^19^, we estimated a 7% death from the disease for the PLHIV population. Individuals were grouped into exposed and non-exposed considering a 2:1 ration, and we adopted a 80% study power, 95% confidence interval (CI), and a 5% alpha error. With that, we reached a sample size qual to 483 patients – 322 in the intervention group and 161 in the control group.

### Randomization

For randomization, we used a series of sealed envelopes containing a computer-generated number of 0 or 1, which were opened sequentially when the patient was included in the study. Randomization sequence, participants registration, and interventions were performed by the project team (coordinator, field coordinator, auxiliaries, nurses, and physicians).

After the first consultation with the nursing staff at the CPH outpatient clinic, patients were invited to participate in the study. On this occasion, patients were informed about the purpose of the study, and those who agreed to participate were requested to sign an informed consent form (ICF). Then, patients were randomized into intervention and control groups.

### Follow-Up

Once randomized into control and intervention groups, participants underwent two monthly follow-up visits at CPH. The last patient entered the study in April 2016, and the final follow-up session was held in October 2016.

Data on mortality and TB diagnosis were obtained from medical records and associated with the Information System for Notifiable Diseases (Sinan TB) and the Mortality Information System (SIM) for the state of Pernambuco using the RecLink III software^11^.

### Statistical Methods

All data analyses were performed using the Stata 15 software. The primary outcome – death from all causes – was determined by survival analysis and Kaplan-Meier estimator, and survival curves were compared using the Z statistics obtained with the log-rank test. Follow-up period was considered as the time between diagnosis and death (failure) or, for surviving cases (censorship), as the time between diagnosis and the completion of the six-month follow-up.

The secondary outcome – TB diagnosis – was obtained by comparing the number of cases notified in both intervention and control group. The accuracy of the screening method for TB was measured based on sensitivity, specificity, positive predictive value (PPV), and negative predictive value (NPV). We also evaluated screening effectiveness for the combination between chest X-ray and clinical algorithm assessing signs and symptoms related to TB, such as fatigue, shortness of breath, presence of lump, or body pain (Table 1). Considering both confirmed and empirical cases, the cumulative incidence of TB was compared between intervention and control group, as well as the cure proportion at the end of the treatment. Case severity was analyzed based on the occurrence of unfavorable outcomes (abandonment and death).

**Table 1:** Prevalence, predictive values, sensitivity, and specificity of TB-related symptoms and other screening factors, Recife, 2014–2016.

TB-related Symptoms	Prevalence in the population	Prevalence among TB cases	Sensitivity	Specificity	PPV	PNV
Cough	30 (25.4–34.9)	71 (52.5–84.9)	70.6 (53.9–83.2)	74.1 (69.2–78.4)	21.2 (14.7–29.7)	96.2 (93.2–97.9)
Fever	18.9 (15.1–23.)	50 (32.4–67.6)	50 (34.1–65.9)	84.2 (80.0–87.7)	23.9 (15.5–35.0)	94.4 (91.3–96.5)
Night sweats	12.8 (9.6–16.6)	29.4 (15.1–47.5)	29.4 (16.8–46.2)	88.9 (85.1–91.8)	20.8 (11.7–34.3)	92.7 (89.3–95.0)
Weight loss	47.9 (42.7–53.1)	82.4 (65.5–93.2)	82.4 (66.5–91.7)	55.6 (50.3–60.7)	15.6 (11.0–21.6)	96.9 (93.5–98.6)
**WHO's Screening**	**56.5 (51.3–61.3)**	**85.3 (68.9–95.0)**	**85.3 (69.9–93.6)**	**46.4 (41.2–51.6)**	**13.6 (9.7–18.9)**	**97.0 (93.1–98.7)**
**Other TB-related symptoms**						
Shortness of breath	18.4 (14.6–22.6)	35.3 (19.7–53.5)	35.3 (21.5–52.1)	83.3 (79.0–86.9)	17.4 (10.2–28.0)	92.8 (89.4–95.2)
Weakness	38.6 (33.6–43.7)	70.6 (52.5–84.9)	70.6 (53.8–83.2)	64.6 (59.4–69.5)	16.6 (11.4–23.5)	95.7 (92.2–97.6)
Chest or back pain	20.7 (16.8–25.2)	44.1 (27.2–62.1)	44.1 (28.9–60.6)	81.6 (77.1–85.3)	19.2 (12.0–29.3)	93.6 (90.3–95.9)
Lump	15.7 (12.2–19.8)	26.5 (12.9–44.4)	26.5 (14.6–43.1)	85.4 (81.2–88.7)	15.3 (8.2–26.5)	92.1 (88.6–94.6)
**Other symptoms combination**						
WHO's screening + chest X-ray	58.6 (53.5–63.6)	85.3 (68.9–95.0)	85.3 (69.9–93.6)	44.0 (38.9–49.3)	13.1 (9.3–18.2)	96.8 (92.7–98.6)
At least 2 WHO's screening	33.0 (28.2–38.0)	82.4 (65.5–93.2)	82.4 (66.5–91.7)	71.9 (66.9–76.4)	22.6 (16.1–30.7)	97.6 (94.9–98.9)
At least 3 WHO's screening	15.4 (11.9–19.5)	54.9 (35.1–70.2)	52.9 (36.7–68.6)	88.3 (84.5–91.3)	31.0 (20.6–43.8)	95.0 (92.0–96.9)
At least 4 WHO's screening	4.8 (2.9–7.5)	8.8 (1.9–23.7)	8.8 (3.0–23.0)	95.6 (92.9–97.3)	16.7 (5.8–39.2)	91.3 (88.0–93.8)
Cough + Fever	1.3 (0.4–3.1)	0 (0–10.3)	0 (0–1.2)	98.5 (96.6–99.4)	0 (0–43.5)	89.6 (86.1–92.3)
Cough + Night sweats	0.5 (0–1.9)	0 (0–10.3)	0 (0–1.2)	99.4 (97.9–99.8)	0 (0–65.8)	90.9 (87.6–93.4)
Cough + Weight loss	9.3 (6.6–12.7)	17.6 (6.7–34.5)	17.7 (8.3–33.5)	91.5 (88.1–94.0)	17.1 (8.1–32.7)	91.8 (88.4–94.3)
Fever + Night sweats						
Fever + Weight loss	4.5 (2.7–7.1)	5.9 (0.7–19.7)	5.9 (1.6–19.1)	95.6 (92.9–97.3)	11.8 (3.3–34.3)	91.1 (87.7–93.6)
Night sweating + Weight loss	1.9 (0.7–3.8)	5.9 (0.7–19.7)	5.9 (1.6–19.1)	98.5 (96.6–99.4)	28.6 (8.2–64.1)	91.3 (88.0–93.8)
Cough + Fever + Night sweats	0.2 (0–1.4)	2.9 (0–15.3)	2.9 (0.5–14.9)	100 (98.9–100)	100 (20.7–100)	91.2 (87.9–93.7)
Cough + Fever + Weight loss	5.6 (3.5–8.4)	29.4 (15.1–47.5)	29.4 (16.8–46.2)	96.8 (94.3–98.2)	47.6 (28.3–67.6)	93.2 (90.1–95.4)
Cough + Night sweats + Weight loss	2.4 (1.1–4.5)	2.9 (0–15.3)	2.9 (0.5–14.9)	97.7 (95.5–98.8)	11.1 (2.0–43.5)	91.0 (87.6–93.5)
WHO's screening + Shortness of breath	15.4 (11.9–19.5)	32.4 (17.4–50.5)	32.4 (19.1–49.1)	86.2 (82.2–89.5)	19.0 (10.9–30.9)	92.8 (89.4–95.1)
WHO's screening + Weakness	32.2 (27.5–37.2)	64.7 (46.5–80.3)	64.7 (47.9–78.5)	71.1 (66.0–75.6)	18.2 (12.3–26.0)	95.3 (92.0–97.3)
WHO's screening + Chest or back pain	18.4 (14.6–22.6)	44.1 (27.2–62.1)	44.1 (28.9–60.6)	84.2 (80.0–87.7)	21.7 (13.6–32.8)	93.8 (90.5–96.0)
WHO's screening + Lump	10.6 (7.7–14.2)	23.5 (10.7–41.2)	23.5 (12.4–40.0)	90.6 (87.1–93.3)	20 (10.5–34.8)	92.3 (88.9–94.7)
WHO's screening + Shortness of breath + Weakness + Chest or back pain + Lump	2.4 (1.1–4.5)	11.8 (3.3–27.5)	11.8 (4.7–26.6)	98.5 (96.6–99.4)	44.4 (18.9–73.3)	91.8 (88.6–94.2)
WHO's screening + At least one signal (shortness of breath or weakness or pain or lump)	41.8 (36.7–46.9)	67.6 (49.5–82.6)	67.7 (50.8–80.9)	60.8 (55.6–65.8)	14.7 (10.0–21.0)	95.0 (91.2–97.2)
WHO's screening considering only cough for more than 2 months	52.4 (47.2–57.5)	85.3 (68.9–95.0)	85.3 (69.9–93.6)	50.9 (45.6–56.1)	14.7 (10.5–20.3)	97.2 (93.6–98.8)

TB: tuberculosis; PPV: positive predictive value; PNV: negative predictive value; WHO: Wolrd Health Organization.

Note: WHO's screening refers to the screening method recommended by the World Health Organization.

We calculated the median time between HIV diagnosis and clinical suspicion of TB; clinical suspicion of TB and TB treatment initiation; and HIV diagnosis and TB treatment initiation. Clinical suspicion was considered as the date corresponding to smear microscopy, sputum culture or GeneXpert examination request or the date indicated in the medical record as TB-related symptoms onset. Cumulative incidence was calculated by dividing confirmed cases by the total number of cases in each group.

The p-value obtained by Chi-square test was used to verify statistically significant differences between the groups. Means were compared using the T-student test, and medians using the Kruskal-Wallis test.

Alpha values equal to 0.05 were considered as statistically significant.

### Ethical Aspects

This study was conducted according with the Guidelines and Regulatory Standards for Research Involving Human Subjects, established by the Resolution No. 466 of December 12, 2012 of the National Health Council^21^. The project was approved by the Research Ethics Committee of the Aggeu Magalhães Research Center, Fiocruz-PE, No. 279.324. The clinical trial was approved by the Brazilian Registry of Clinical Trials (ReBEC), under registration number RBR-22t943.

## RESULTS

**Figure 1:**
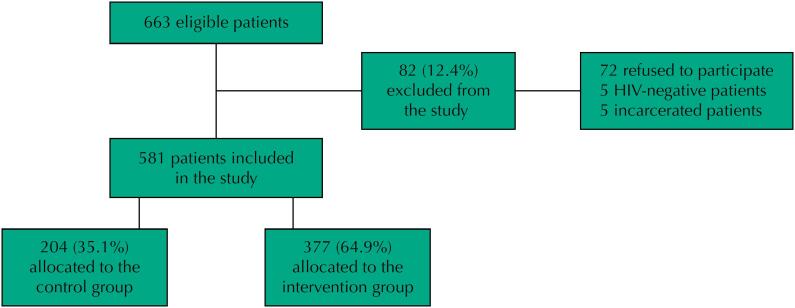
Research flowchart.

Among the 663 eligible patients admitted to the Correia Picanço Hospital (CPH) during the study period, 82 (12.4%) were excluded – 72 (87.8%) of whom refused to participate and 10 (12.2%) who were excluded for presenting negative HIV test results (n = 5) or for being incarcerated (n = 5). Thus, our study sample comprised 581 patients: 377 (64.9%) in the intervention group and 204 (35.1%) in the control group (routine) (Figure 1).

Regarding sample characterization, most participants were male (71.8%), with a mean age of 35 years (minimum 18 and maximum 71 years); 63.3% were residents in Recife, the state capital, and almost 10% were illiterate and presented with a low CD4 count. Socioeconomic and clinical characteristics were similar in both groups, indicating the efficacy of the randomization.

Clinical algorithm-based screening for tuberculosis (TB) showed a moderate sensitivity (85.3%) and low specificity (46.4%), reaching a very low positive predictive value (PPV; 13.6%), but a high negative predictive value (NPV; 97%). Amongst the various symptom combinations tested, that recommended by the World Health Organization (WHO) provided the most satisfactory results – although not considered excellent. Screening performance remained the same after chest X-ray inclusion, with a 44% specificity, a 13.1% PPV, and a 96.8% NPV (Table 1).

We identified 53 cased of TB during follow-up, 34 in the intervention group and 19 in the control group. Both groups showed similar TB cumulative incidences: 9% for the intervention (95% CI: 6.3%–12.4) and 9.3% for the control (95% CI: 5.7%–14.2%) (p = 0.906).

We detected seven (20.6%) bacteriologically confirmed TB cases in the intervention group and two (10.5%) in the control group, showing a statistically non-significant difference (p = 0.297). Pulmonary TB was the predominant form of the disease in both groups, especially the intervention group (52.9% *vs*. 42.1% in the control group), but without significant difference (p = 0.748). A “cure” outcome occurred in ten (30.3%) of the 34 confirmed cases of TB in the intervention group and in nine cases of the control group (50%), without statistically significant difference (p = 0.454) (Table 2).

**Table 2:** Characteristics of tuberculosis (TB) cases, detection, and death between Intervention and Control groups, Recife, 2014–2016.

		Intervention group	Control group	p
		n (%)	n (%)	
Tuberculosis				
	No	343 (91.0)	185 (90.7)	0.906
	Yes	34 (9.0)	19 (9.3)	
	Total	377	204	
Form of TB				
	Pulmonary	18 (52.9)	8 (42.1)	
	Extrapulmonary	9 (26.5)	6 (31.6)	0.748
	Disseminated	7 (20.6)	5 (26.3)	
	Total	34	19	
Confirmed Tuberculosis				
	Yes	7 (20.6)	6 (31.6)	0.372
	No	27 (79.4)	13 (68.4)	
	Total	34	19	
TB Outcome				
	Cure	10 (30.3)	9 (50.0)	0.164
	Others	23 (69.7)	9 (50.0)	
	Total	33	18	
Death				
	No	351 (93.1)	194 (95.1)	0.341
	Yes	26 (6.9)	10 (4.9)	
	Total	377	204	
TB cases Characteristics				
	Time in days of diagnosis (mean)	49.9	54.6	0.967
Exams performed				
	Sputum smear	17 (81.0)	4 (19.0)	0.228^a^
	GeneXpert	3 (60.0)	2 (40.0)	0.400^a^
	Culture	7 (87.5)	1 (12.5)	0.875^a^
	Chest X-ray	11 (64.7)	6 (35.3)	0.353^a^
	Total	38	13	
Positive exams				
	Sputum smear	2 (50.0)	2 (50.0)	0.228^a^
	GeneXpert	3 (100.0)	0 (0.0)	0.400^a^
	Culture	1 (100.0)	0 (0.0)	0.875^a^
	Chest X-ray	5 (31.3)	1 (16.7)	0.353^a^
	Total	11	3	
Diagnostic site				
	CPH	26 (68.4)	12 (31.6)	0.358^a^
	Other services	5 (55.6)	4 (44.4)	
	Total	31	16	
Location of diagnosis in CPH				
	Outpatient care	17 (70.8)	7 (29.2)	0.602^a^
	Inpatient care	9 (69.2)	4 (30.8)	
	Total	26	11	
ART				
	Yes	28 (66.7)	14 (33.3)	0.342^a^
	No	6 (54.6)	5 (45.5)	
	Total	34	19	

CPH: Correia Picanço Hospital; ART: antiretroviral therapy.

a p-value of the chi-square test or exact Person test.

Both groups showed a similar median time between HIV diagnosis and clinical suspicion of TB, with one day in the intervention group (IQR = 0–5 days) and 25 days in the control group (IQR = 5.5–69) (p = 0.093).

The median time between clinical suspicion of TB treatment initiation was 29 days (IQR = 14–75 days) for the intervention group and 51.5 days (IQR = 7.5–122) for the control group (p = 0.864).

Likewise, the median time between HIV diagnosis and TB treatment initiation was similar between groups, with 40 days (IQR = 18–77) in the intervention group and 50 days (IQR = 15–75 days) in the control group, without statistically significant difference (p = 0.978).

A total of 36 deaths occurred during the follow-up period – 26 (6.9%) in the intervention group and 10 (4.9%) in the control group (p = 0.341), – with an overall cumulative mortality equal to 62 deaths per 1,000 inhabitants. Corresponding cumulative mortality was 69 per 1,000 inhabitants in the intervention group and 49 per 1,000 inhabitants in the control group (p = 0.341).

Ten of the 36 deaths recorded in the study were related to TB – six in the intervention and four in the control group. Thus, TB mortality rate was 17.2 per 1,000 inhabitants in the overall study population, 18.6 per 1,000 in the intervention group, and 14.7 per 1,000 in the control, without statistical difference (p = 0.705). Treatment abandonment was also similar between groups, with 21% in the intervention and 18% in the control (p = 0.761).

**Figure 2:**
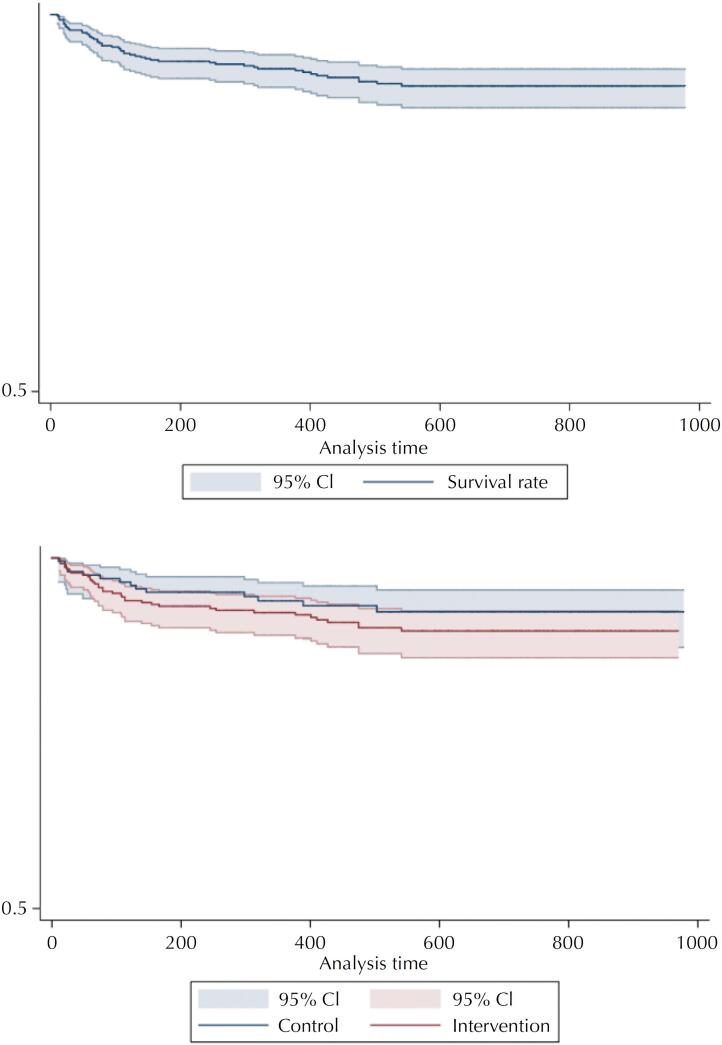
Kaplan-Meier curve for the overall survival probability in the first six months after HIV diagnosis.

The follow-up time for the entire cohort was 175.9 days. Total person-years at risk was equal to 266, and the all-cause mortality rate was 1.4 per 100 person-years. Figure 2 presents the Kaplan-Meier Curve for the probability of overall survival in the first six months after HIV diagnosis, reaching a 94% probability by the end of follow-up. Regarding the values for each group, mean follow-up time was 175 days for the intervention and 177.4 for the control; total person-years at risk was 172.3 for the intervention group and 93.7 for the control; and mortality rate was 15.1 per 100 person-years for the intervention and 10.7 per 100 person-years for the control. The Kaplan-Meier probability of overall survival in the first six months was 93.1% in the intervention and 95.1% in the control group, without statistically significant differences from the log-rank test (p = 0.343) (Figure 2).

## DISCUSSION

This pragmatic clinical trial demonstrates that the clinical algorithm-based screening method for tuberculosis (TB) recommended by the World Health Organization (WHO) shows moderate sensitivity and low specificity. Intervention and control group showed no differences regarding TB cases detection and outcomes (abandonment and death), as well as for the overall survival.

The screening method reached a high negative predictive value (NPV) – an important factor for clinical care given that HIV-infected patients who report no cough, fever, night sweats, and weight loss most likely do not present with TB (97%).

Our results also indicate that the period between HIV diagnosis, clinical suspicion of TB, TB treatment initiation were lower in the intervention group when compared to the control group. If we were able to properly certify the dates of clinical suspicion of TB in the control group, these differences could be greater. Few TB-related tests were requested for participants in this group, and we verified the lack of specific registry referring to suspected TB in most medical records. Some cases did contain notes on the presence of cough, fever, or weight loss symptoms, but no record of requested tests. In this sense, standardizing the record of TB-related symptoms would lead physicians to standardize the search for TB.

According to the WHO, the time interval between clinical suspicion of TB and treatment initiation could have been even shorter in the intervention group if GeneXpert MTB/RIF exams were performed at the health facility^9^. A study conducted in South Africa showed that GeneXpert MTB/RIF delays speeds up TB diagnosis when compared to the standard procedure (smear microscopy)^22^.

Although several studies have evaluated TB screening procedures in people living with HIV (PLHIV) based on the guidelines established by the WHO^9^^–^^17^, none of these were conducted exclusively in Brazil. In this sense, analyzing different scenarios with different prevalence is extremely important for characterizing the effectiveness of screening for TB in different populations^9^, especially because PPV and NPV may change according with changes in the prevalence of TB.

The main indicators of TB screenings for population are sensitivity and specificity; in turn, PPV and NPV are the major focus in clinical practice. The values found in our study population are similar to those reported in a study conducted by Getahun et al. (2011)^9^ – NPV equal to 97.7% for a 5% prevalence. However, other studies found lower PPV for TB screening^10^. High NPV values contribute to one of the goals proposed by the WHO, namely ruling out active TB disease for initiating preventive treatment^9^.

Several studies found unacceptable sensitivity and specificity values for symptom-based TB screening in certain scenarios, especially regarding the number of false positives due to comorbidities including similar symptoms.^9^^,^^13^^,^^14^^,^^17^ These findings reinforce the need for a more effective TB screening in PLHIV. In Brazil, specialized health services for HIV/Aids have highly-trained professionals who follow the guidelines established by the Ministry of Health and made available through manuals and regulations incorporated into health service routines^24^.

Most studies approaching TB screening sought to evaluate only bacteriologically confirmed cases – either by sputum smear microscopy, sputum culture, or GeneXpert^10^^,^^11^^,^^13^^,^^15^^,^^17^. Considering that analyzing solely confirmed cases would restrict the study sample to individuals able to provide sputum, and acknowledging that PLHIV produce and release little sputum, we used TB screening to detect both confirmed and presumed cases of TB. Moreover, a study based on the positivity of bacteriology would be unfeasible before the insufficient structure of the CPH to induct sputum release.

As increasing cases of presumptive TB have been reported worldwide, accounting for nearly half of the total cases, we sought to analyze TB screening amongst presumptive TB, thus requiring screening methods based on clinical-epidemiological criteria^24^. In severe cases, TB treatment is often initiated without tests results. Although some studies have shown that other symptoms may be related to extrapulmonary TB, studies on screening effectiveness often focus primarily on pulmonary TB^9^. Given that extrapulmonary TB is responsible for around 15% of cases in PLHIV worldwide and that in some regions this value exceeds 20%, screening methods should consider extrapulmonary TB.

Different from previous studies on the topic, our study considered the impact of screening for TB treatment outcomes. However, we found no significant differences in the mortality rate (nor in other treatment outcomes) for both intervention and control groups. This finding may be explained by the low impact of screening in the routine of the service or by the organization of its healthcare team, which includes trained professionals who follow the recommendations established by the WHO.

Our study has some limitations. Firstly, the infrastructure of the Correia Picanço Hospital (CPH) lacked medical devices for performing chest X-rays (which could have positively impacted screening) and GeneXpert test, thus delaying cases confirmation. Confirmed cases of tuberculosis may also have been underreported by the Information System for Notifiable Diseases (Sinan), which we attempted to circumvent by using the state-wide database. Moreover, the infectologists were divided by study group, so that it was not possible to blind them.

On the other hand, our results advance knowledge to the study field. By evaluating the effectiveness of TB screening through a pragmatic clinical trial, we verified that performing an additional interview for detecting TB at every follow-up visit to the tertiary service is not necessary. Rather, attending physicians could incorporate this procedure into their routine care at the specialized services for PLHIV. Considering that other tertiary services for HIV/AIDS in the country are likely to present the same reality, our findings may be generalized for them.
